# Glucocorticoid receptor inhibits Th2 immune responses by down-regulating Pparg and Gata3 in schistosomiasis

**DOI:** 10.3389/fimmu.2025.1518586

**Published:** 2025-03-24

**Authors:** Tao Sun, Xiaojuan Bi, Ning Yang, Xue Zhang, Jin Chu, Liang Li, Hui Liu, Rui Tang, Renyong Lin

**Affiliations:** 1 State Key Laboratory of Pathogenesis, Prevention and Treatment of High Incidence Diseases in Central Asia, Clinical Medical Research Institute, The First Affiliated Hospital of Xinjiang Medical University, Urumqi, Xinjiang, China; 2 Department of Tropical Infectious Diseases, Naval Medical University, Shanghai, China

**Keywords:** glucocorticoid receptor, schistosomiasis, Pparg, Gata3, Th2 immune responses

## Abstract

**Introduction:**

The Th2 immune response plays a pivotal role in the pathogenesis of schistosomiasis, contributing to the formation of hepatic granulomas and fibrosis. While the glucocorticoid receptor (GR) is a ubiquitously expressed nuclear receptor that mediates anti-inflammatory effects, its impact on Th2 responses in schistosomiasis remains underexplored. Thus, this study aimed to investigate the potential impact of GR activation on the hepatic Th2 immune response in schistosomiasis using the synthetic glucocorticoid dexamethasone.

**Method:**

*In vivo*, *Schistosoma japonicum*-infected mice were treated with dexamethasone, while in vitro studies were conducted on Th2 cells. Additionally, RNA sequencing and single-cell sequencing were integrated to identify key transcription factors influenced by GR activation during Th2 cell differentiation, with gene expression levels validated both in vivo and in vitro.

**Results:**

*In vivo*, GR activation markedly reduced the size of *Schistosoma egg* granulomas and substantially repressed the transcription of key Th2-related cytokines, such as IL-4, IL-5, and IL-13. *In vitro*, GR activation inhibited the transcription of IL-4, IL-5, and IL-13, as well as the secretion of IL-4 in Th2 cells. An integrated analysis of RNA sequencing and single-cell sequencing revealed that GR activation downregulated the expression of two major transcription factors, Gata3 and Pparg, which were elevated in infected mouse livers and Th2 cells but decreased following dexamethasone treatment.

**Conclusion:**

GR activation may suppress the Th2 immune response triggered by egg antigens by downregulating the expression of the key transcription factors Gata3 and Pparg. While these findings provide insights into a potential complementary therapeutic strategy, further research is necessary to assess the feasibility and safety of targeting GR activation for the treatment of schistosomiasis.

## Introduction

Schistosomiasis is a zoonotic parasitic disease with a widespread global distribution, representing a significant threat to public health. It is the second most prevalent parasitic disease worldwide, primarily affecting regions in China, the Philippines, and Sulawesi Island in Indonesia (1, 2). According to the World Health Organization (WHO), over 230 million individuals currently suffer from schistosomiasis, resulting in approximately 200,000 deaths annually (3, 4). In the life cycle of *Schistosoma*, humans serve as the definitive host, while freshwater snails act as the intermediate host, playing a critical role in disease transmission (5). Infection occurs following schistosome cercariae in contaminated water penetrating the skin or mucous membranes and subsequently entering the bloodstream. The schistosomula then migrate through the circulatory system and are ultimately transported into the splanchnic arteries, including the celiac and mesenteric arteries, before eventually reaching the liver (6, 7). Unable to traverse the hepatic sinusoids, schistosomula reside within the branches of the hepatic portal vein, where they feed on blood and mature. Upon maturation, the worms pair and migrate to their preferred sites for egg-laying (8–10). Notably, each female worm can lay between 300 and 3,000 eggs daily, with the majority deposited in the liver and intestinal wall and the remaining eggs excreted from the body. The soluble egg antigen (SEA) triggers a robust immune response, activating hepatic stellate cells (HSCs) and inducing their differentiation into proliferative, contractile, and fibrogenic myofibroblasts (11, 12). This process results in the accumulation of extracellular matrix (ECM) proteins, which replace damaged liver tissue, ultimately culminating in hepatic fibrosis (13, 14). At present, praziquantel is the only clinically effective drug approved for the treatment of schistosomiasis (15). However, due to its suboptimal efficacy and the risk of drug resistance, there is a pressing need to develop more cost-effective alternatives or complementary treatments for schistosomiasis (16, 17).

The Th2 immune response, initiated by soluble egg antigens released from liver-trapped eggs, plays a vital role in regulating disease progression in schistosomiasis (18, 19). Cytokines generated during the Th2 response facilitate B cell proliferation, immunoglobulin class-switching to IgE, eosinophilia, mast cell activation, goblet cell hyperplasia, macrophage polarization towards an M2-like phenotype, and smooth muscle contraction (20, 21). During the initial stages of schistosomiasis, the Th2 response triggered by egg antigens protects the host by downregulating the expression of pro-inflammatory mediators associated with the Th1 immune response activated by parasite antigens (22). However, sustained activation of the Th2 response may lead to excessive hepatic fibrosis, which contributes to chronic morbidity (23). Consequently, the suppression of the persistent Th2 immune response has emerged as a vital therapeutic strategy for the management of schistosomiasis.

The glucocorticoid receptor (GR) is a nuclear receptor widely expressed across various human cell types, playing an instrumental role in the regulation of carbohydrate, fat, and protein metabolism, as well as in the modulation of anti-inflammatory and immune responses (24, 25). Glucocorticoids (GCs), which act as agonists of GRs, bind to the receptor in the cytoplasm, promoting its activation and subsequent translocation to the nucleus, where it regulates gene transcription (26). Clinically, GCs have been employed for decades for the management of liver failure by activating GRs, primarily owing to their capacity to rapidly suppress excessive inflammatory and immune responses (27, 28). Moreover, GCs have shown efficacy in the treatment of conditions such as systemic lupus erythematosus and asthma exacerbations through GR activation, which suppresses the expression of type 2 cytokines (29, 30). However, the effects of GR activation on the Th2 immune response in schistosomiasis remain elusive. This study unveiled that GR activation effectively suppressed the hepatic Th2 immune response in schistosomiasis by downregulating the expression of the key transcription factors Gata3 and Pparg, offering valuable insights into the mechanism of Th2 immune response inhibition through GR activation and may present a novel complementary therapeutic approach for the management of schistosomiasis.

## Methods and materials

### Animals

Male C57BL/6 mice, aged 6 to 8 weeks, were procured from SLAC Laboratory in Shanghai, China, and were randomly assigned to three groups (1): Uninfected + Vehicle group, (2) Infected + Vehicle group, and (3) Infected + Dex group (n = 6 per group). All animal experiments were conducted in accordance with the National Institutes of Health’s Guidelines for the Care and Use of Laboratory Animals, and this study was approved by the Animal Ethics Committee of Naval Medical University (access number SCXK [Shanghai] 2022-0009). To establish a mouse model of schistosomiasis, mice were percutaneously exposed to 25 *S. japonicum* cercariae released from laboratory infected Oncomelania hupensis snails, which were sourced from the National Institute of Parasitic Disease at the Chinese Center for Disease Control and Prevention (Shanghai, China). From days 21 to 42 post-infection, mice in the dexamethasone group were intraperitoneally injected with 1 mg/kg dexamethasone (MK-125, Selleck, Shanghai, China) every other day, while the vehicle-treated groups were administered an equivalent volume of the corresponding vehicle. Mice were euthanized at the end of the treatment period for further analysis (**Supplementary Figure S1**).

### *In vitro* mouse primary T-cell polarization

Naive CD4^+^ T cells were purified from C57BL/6 mice and cultured for T-helper (Th) cell differentiation as described in earlier studies (31, 32). Briefly, CD4^+^ T cells were isolated from the spleens of C57BL/6 mice using a Naive CD4^+^ T Cell Isolation Kit (#130-117-043, Miltenyi Biotec, Bergisch Gladbach, Germany) according to the manufacturer’s instructions. Next, naive CD4^+^ T cells (1×10^6^) were plated in a 24-well plate precoated with 1 µg/mL anti-CD3e antibody (Cat# 100340, Biolegend) and 1 µg/mL anti-CD28 antibody (Cat# 102116, Biolegend). Th2 cell differentiation was induced by culturing cells in the presence of 20 ng/mL murine IL-2 (Cat# 714604, Biolegend), 10 ng/mL murine IL-4 (404-ML, R&D Systems), 10 µg/mL anti-IL-12 (Cat# 505308, Biolegend), and 10 µg/mL anti-interferon (IFN)-γ (Cat# 505834, Biolegend) for 3 days. Afterward, 2 µM dexamethasone was added, and the cells were incubated for 24 hours before cytokine expression was assessed.

### RNA sequencing and analysis

Total RNA was extracted using TRIzol^®^ reagent (Invitrogen^®^ 15596018) following the manufacturer’s protocol. Following RNA extraction and purification, RNA-seq libraries were constructed using the Illumina Paired-End Sample Prep kit and sequenced on the Illumina NGS platform. The obtained high-quality reads were aligned to the Mus musculus GRCm38 reference genome using STAR. Based on the alignment results, gene-level read counts were determined using Kallisto, and differentially expressed genes (DEGs) were identified using the DESeq2 algorithm with the following criteria: |log2FC| > 1 and *P*-value < 0.05. DEGs were selected for further functional analysis, including pathway enrichment, protein-protein interaction, and regulatory network analyses. Gene Ontology (GO) and Kyoto Encyclopedia of Genes and Genomes (KEGG) enrichment analyses were performed using the R package clusterProfiler. Protein-protein interaction (PPI) networks were constructed using STRING (https://string-db.org). Transcription factors were identified based on the transcriptional regulatory relationships documented in the TRRUST database (https://www.grnpedia.org/trrust/).

### Public data sources

In the present study, the public single-cell RNA-seq dataset (GSE220286) was downloaded from the Gene Expression Omnibus (GEO, https://www.ncbi.nlm.nih.gov/). Raw gene expression matrices were imported into R (version 4.2.2) and converted to Seurat objects using the Seurat R package (version 5.0.1) (33). The percentage of mitochondrial genes was calculated using the PercentageFeatureSet function, and the data were normalized using the NormalizeData function. Highly variable genes were identified using the FindVariableFeatures function. Principal Component Analysis (PCA) was performed using the RunPCA function, and the top 30 principal components were utilized to facilitate dimensionality reduction and clustering analysis using Uniform Manifold Approximation and Projection (UMAP). Clusters were annotated based on the expression of known marker genes and visualized using the integrated tools within the Seurat package. SCENIC analysis was performed to infer transcription factor regulatory networks from the single-cell RNA-seq data, utilizing the cisTarget databases via the RcisTarget package (34). Cytoscape software was used to visualize the transcription factor-target gene regulatory networks (35).

### Immunohistochemistry

For immunohistochemical analysis, tissue sections were deparaffinized and rehydrated. Endogenous peroxidase activity was then blocked using a 3% H2O2 solution. Antigen retrieval was performed with citrate buffer. To reduce nonspecific binding, sections were incubated with 10% goat serum. Subsequently, they were incubated overnight at 4°C with primary antibodies, including anti-GATA3 (ab199428, Abcam,1:500) and anti-PPARG (WL01800, WanLeiBio, 1:200). The next day, sections were treated with secondary antibodies(ab6721, Abcam, 1:1000), followed by visualization with a DAB staining kit(Abcam), counterstaining with hematoxylin, and sealing with neutral gum.

### Western blotting

Protein expression levels were determined by Western blot analysis. Briefly, total protein from cells was extracted using RIPA lysis buffer supplemented with protease inhibitors and phosphatase inhibitors to prevent protein degradation and dephosphorylation(Solarbio). Protein concentration was measured using the BCA Protein Assay Kit(Pierce). Next, Equal amounts of protein (20μg) for each sample was loaded onto a 10% SDS-PAGE gel and separated. The proteins were then transferred to polyvinylidene difluoride (PVDF) membranes. The membranes were blocked for 1 hour at room temperature with 5% non-fat milk in Tris-buffered saline containing 0.1% Tween-20 (TBST). Following this, the membrane was incubated overnight at 4°C with primary antibodies: anti-GATA3 (ab199428, Abcam, 1:1000), anti-PPARG (WL01800, WanLeiBio, 1:500), or anti-GAPDH (Abcam, ab181602, 1:10000). The secondary antibody used was goat anti-rabbit IgG (Bioss, bs-0295G, 1:1000).

### Quantitative real-time PCR

cDNA synthesis was performed using the HiScript II 1st Strand cDNA Synthesis Kit (R211-01, Vazyme Biotech Co.). Quantitative PCR (qPCR) was performed with primers specific for IL-4, IL-5, IL-13, Gata3, Pparg, and Gapdh, which were synthesized by the Beijing Genomics Institute (BGI) (see **Supplementary Table S1**). ChamQ Universal SYBR qPCR Master Mix (Q711-02, Vazyme Biotech Co.) was employed for the qPCR assays. Relative gene expression levels were calculated using the 2^−ΔΔCt^ method, with GAPDH serving as the internal control for normalization.

### Pathologic features of the liver

To assess the size of hepatic egg granulomas, liver tissue sections were stained with Mayer’s hematoxylin and eosin (H&E). The maximum (longest) and minimum (shortest) diameters of each egg granuloma were measured under a microscope, and the number of granulomas was counted per section. The area of each granuloma was approximated as the product of the maximum and minimum diameters (Area = maximum diameter × minimum diameter). The total granuloma area for each liver sample was calculated as the sum of all granuloma areas, and the average granuloma area per sample was determined by dividing the total area by the number of granulomas.

For fibrosis evaluation, liver sections were stained with Masson’s trichrome. The degree of fibrosis was quantified by calculating the fibrosis score based on the color intensity and area of collagen fibers in the section. Both the color intensity and area of collagen fibers were graded on a scale from 1 to 4. The final fibrosis score for each sample was calculated by multiplying the color grade by the area grade.

All pathological features were evaluated by two pathologists in a blinded manner. The assessors were unaware of the sample group assignments, minimizing potential bias.

### Flow cytometry

Single-cell suspensions were prepared and adjusted to a concentration of 1×10^6^ cells in 100 µL. For surface staining, cells were first incubated on ice with anti-CD16/CD32 antibody (Cat# 100340, Biolegend) to block Fc receptors, followed by staining with APC-conjugated anti-mouse CD4 antibody (Cat# 100516, Biolegend). For intracellular cytokine staining, cells were stimulated for 4 hours using a cell activation cocktail containing Brefeldin A (Biolegend) to inhibit cytokine secretion. After stimulation, they were fixed with Fixation Buffer (Biolegend) and permeabilized using Permeabilization Wash Buffer (Biolegend). Intracellular IL-4 and IFN-γ was stained using PE-conjugated anti-mouse IL-4 antibody (Cat# 504104, Biolegend) and PE-conjugated anti-interferon (IFN)-γ (Cat# 505834, Biolegend). Data acquisition was performed on a FACS Calibur flow cytometer, and analysis was conducted using FlowJo software.

### Statistical analysis

GraphPad Prism software version 9.3.1 was used for statistical analysis. Data were expressed as the mean ± standard deviation (SD). Student’s t-test was used to assess differences between two groups, and one-way ANOVA followed by Tukey post-test was used for comparisons among three or more groups. P-values less than 0.05 were considered statistically significant.

## Results

### GR suppresses the egg antigen-induced Th2 response in schistosomiasis

**Figure 1:**
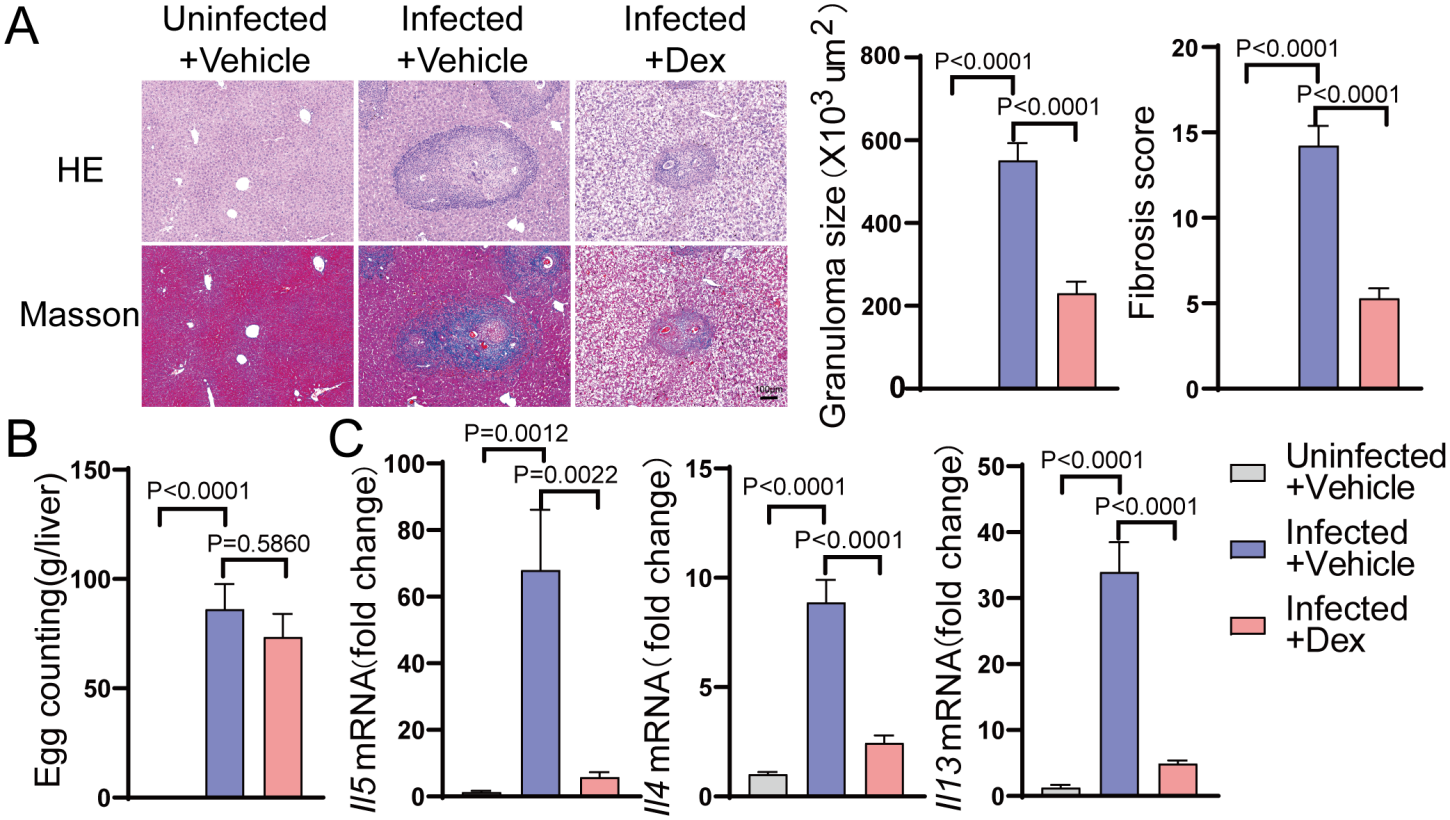
GR activation inhibits the Th2 immune response in the liver of *S.japonicum*-infected mice. **(A)** Size of granulomas and fibrosis scores in the liver (n=6). 200X magnification, scale bar = 100 μm. **(B)** Egg counts (n=6). **(C)** mRNA expression levels of Il4, Il5, and Il13 in the liver (n=6). Data are presented as mean ± SD.

To investigate the effect of GR activation on the hepatic Th2 response in schistosomiasis, dexamethasone was administered to mice infected with *Schistosoma*. Egg counting, HE staining, Masson staining, and qPCR were employed to assess the degree of liver fibrosis and the levels of Th2 cytokines in schistosomiasis. As anticipated, the egg burden was comparable between the Infected + Vehicle group and the Infected + Dex group (**Figure 1B**). HE staining revealed that, compared to the Uninfected + Vehicle group, the number of egg granulomas was significantly higher in the liver of the Infected + Vehicle group. In contrast, the average granuloma size in the liver was significantly lower in the Infected + Dex group. At the same time, Masson staining illustrated extensive collagen deposition within egg granulomas, with fibrosis scores significantly higher in the Infected + Vehicle group compared to controls, whereas these scores were markedly lower in the Infected + Dex group (**Figure 1A**). Additionally, qPCR results indicated that the expression levels of type 2 effector cytokines, including IL-4, IL-5, and IL-13, were significantly higher in the Infected + Vehicle group compared to the control group and significantly lower in the Infected + Dex group (**Figure 1C**). Taken together, these findings suggest that GR activation can suppress the Th2 immune response and mitigate schistosomiasis-associated liver fibrosis.

### GR suppresses the expression of type 2 cytokines in Th2 cells

**Figure 2:**
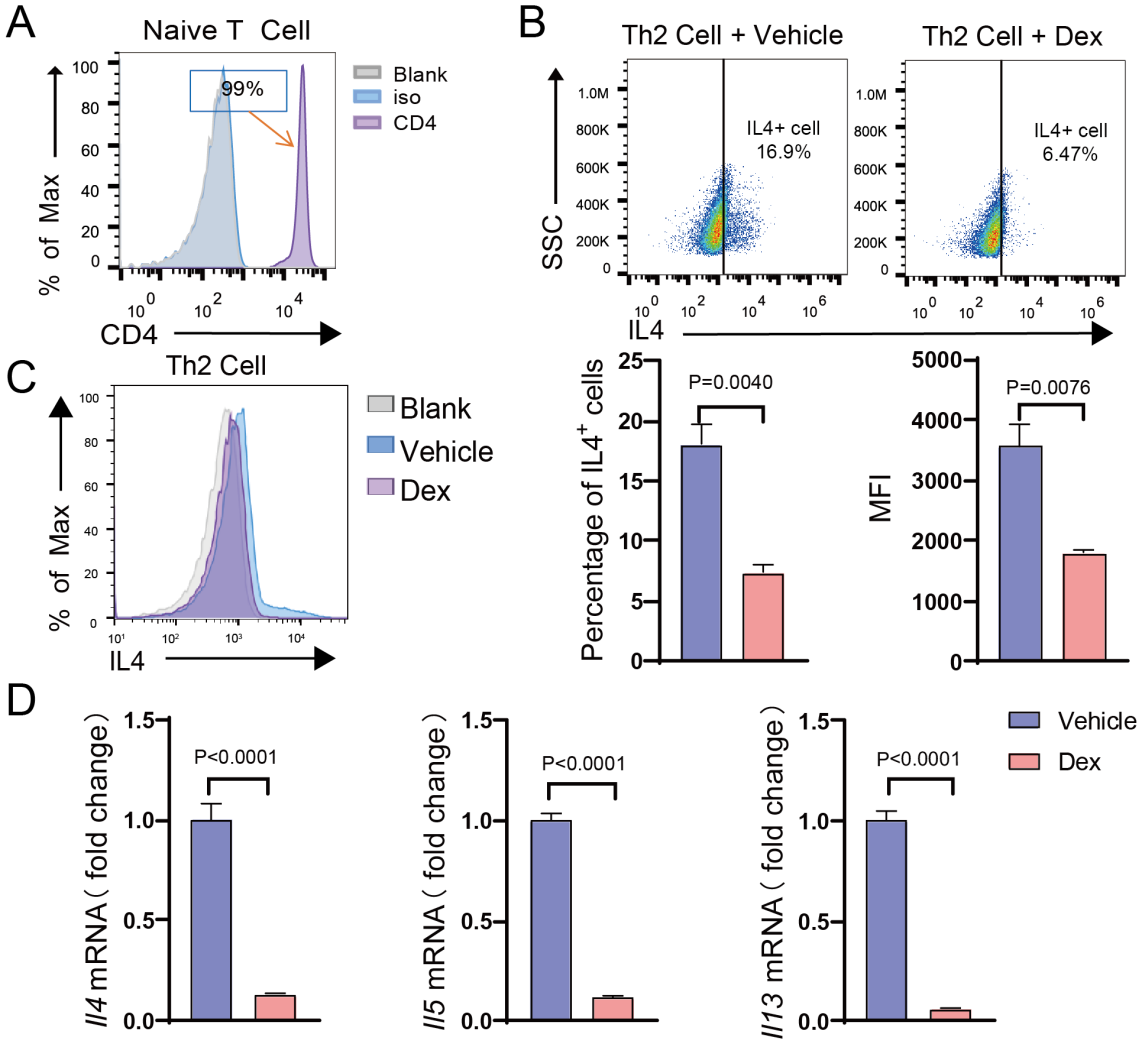
GR activation suppresses the function of Th2 cells. **(A)** Histogram showing CD4 expression (MFI) on naive CD4+ T cells isolated from the spleen (n=3). **(B)** Dot plot showing the gating strategy based on IL-4 in Th2 cells after DEX treatment (n=3). **(C)** Flow cytometry analysis of the proportion of IL-4+ cells and the MFI of IL-4 in Th2 cells after DEX treatment (n=3). **(D)** mRNA expression levels of Il4, Il5, and Il13 in Th2 cells after DEX treatment (n=3). Data are presented as mean ± SD.

Th2 cells play an essential role in type 2 immune responses. To further investigate the role of GR in Th2 cells, splenic CD4^+^ naive T cells were isolated and induced to differentiate into Th2 cells *in vitro*, followed by treatment with dexamethasone. Flow cytometry analysis demonstrated that the purity of the extracted CD4^+^ T cells reached 99% (**Figure 2A**). After *in vitro* induction, the number of IL-4-positive cells significantly increased, indicating the successful polarization of Th2 cells (**Supplementary Figure S4**). Following dexamethasone treatment, qPCR analysis exposed that the mRNA expression levels of type 2 cytokines, including IL-4, IL-5, and IL-13, were decreased in the Dex-treated group (**Figure 2D**). Additionally, the flow cytometry result confirmed a significant reduction in both the proportion of IL-4-positive cells and the mean fluorescence intensity of IL-4 compared to the control group (**Figures 2B, C**). These findings collectively suggest that GR activation significantly inhibits Th2 cell function, leading to a reduction in the translation and secretion of type 2 cytokines.

### Effects of GR on gene expression profile in Th2 cells

**Figure 3:**
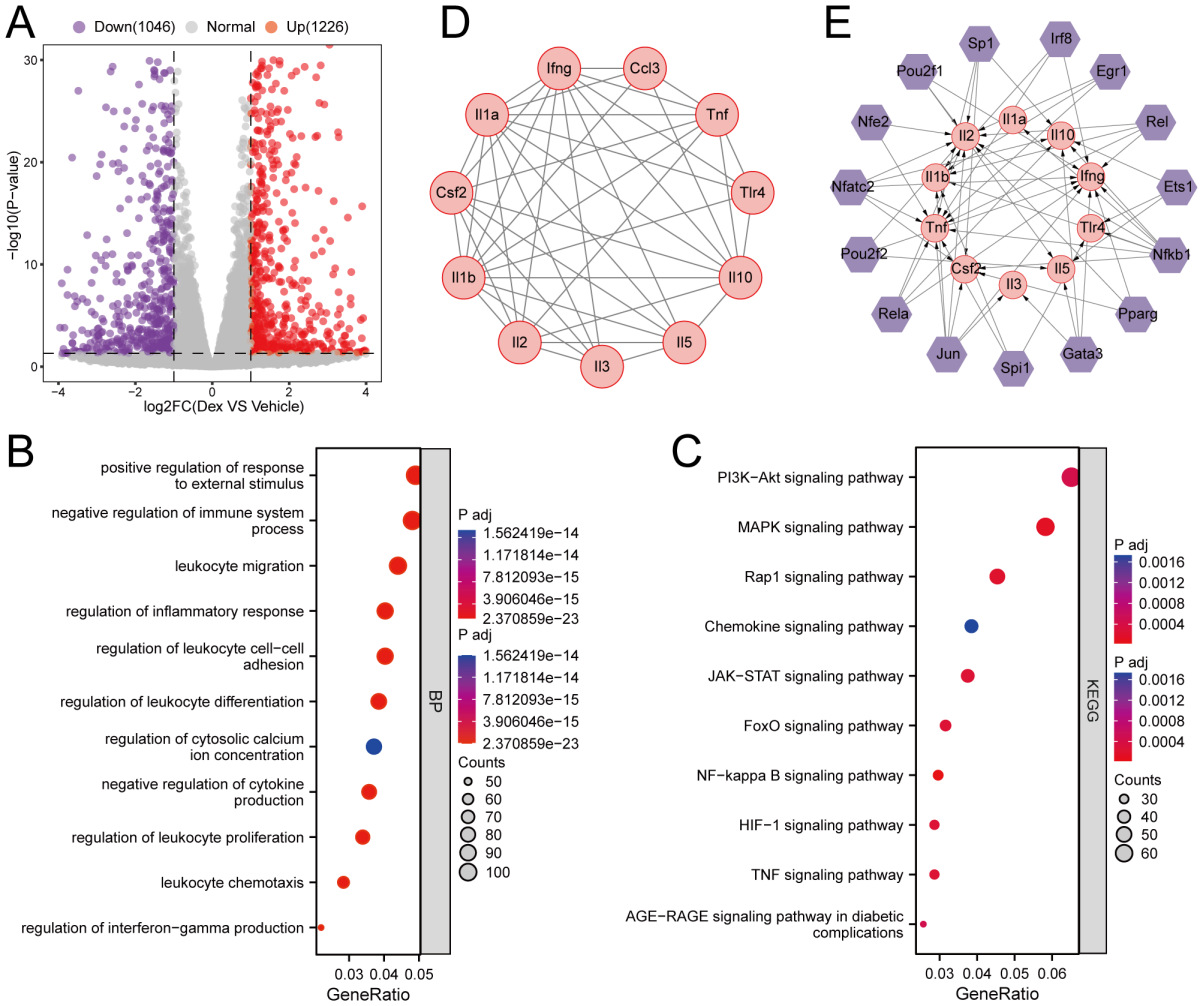
Effects of GR activation on gene expression profile in Th2 cells. **(A)** Volcano plots. The purple and red points represent differentially expressed genes. **(B, C)** KEGG pathways analysis and GO analysis of differentially expressed genes. **(D)** PPI of genes in the clustering module 1. **(E)** The TFs of the clustering module 1 based on the TRRUST database.

To identify key regulatory genes influenced by GR activation, global gene expression profiling was carried out using RNA-seq on dexamethasone-treated Th2 cells. Differential expression analysis yielded a total of 2,272 DEGs compared to the control group, with 1,226 upregulated and 1,046 downregulated genes (**Figure 3A**). GO analysis indicated significant enrichment in biological processes such as regulation of leukocyte differentiation, regulation of inflammatory response, negative regulation of cytokine production, positive regulation of response to external stimulus, and negative regulation of the immune system process (**Figure 3B**). Meanwhile, KEGG pathway analysis highlighted the involvement of several key signaling pathways, including the NF-kappa B signaling pathway, MAPK signaling pathway, Rap1 signaling pathway, and TNF signaling pathway (**Figure 3C**). Afterward, a protein-protein interaction (PPI) network was constructed using the STRING database, and MCODE analysis identified three significant gene modules. Specifically, module 1 was enriched for genes involved in lymphocyte differentiation, while modules 2 and 3 were associated with ribosome biogenesis and phospholipid metabolism, respectively (**Figure 3D**, **Supplementary Figures S2A-C**). Based on the TRRUST database, 15 transcription factors that may regulate the expression of genes in Module 1 were identified, and a TF-mRNA regulatory network was generated (**Figure 3E**).

### Key transcription factors that induce Th2 differentiation in schistosomiasis

**Figure 4:**
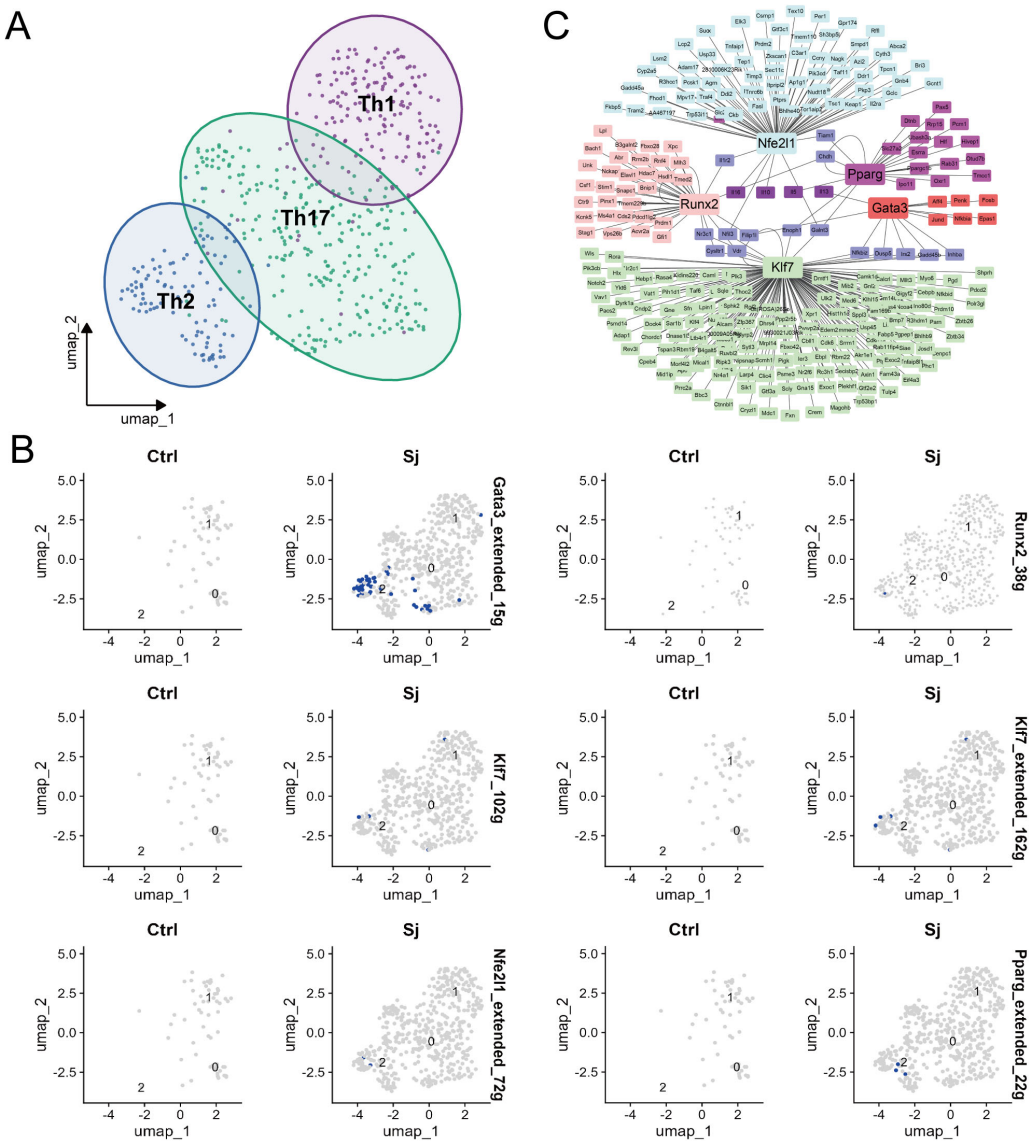
Identification of transcription factors for Th2 cell differentiation in *S.japonicum*-infected mice. **(A)** UMAP plot depicting cells derived from CD4 T cells and the identified clusters. **(B)** UMAP plots of cells visualizing binding motif activity in Ctrl and Sj samples. **(C)** Expression regulatory network of TFs.

To examine transcription factors that drive Th2 differentiation in response to schistosome egg granulomas, cell-type annotation was conducted, and the SCENIC software package was used to identify key regulons from single-cell RNA-seq data (**Supplementary Figures S3A-E**). Our analysis revealed three distinct cell populations corresponding to Th1, Th2, and Th17 cells (**Figure 4A**). The SCENIC workflow comprises three primary stages, namely coexpression analysis, motif enrichment for target genes, and regulon activity scoring. Utilizing this methodology, five key regulons associated with the transcription factors Gata3, Pparg, Nfe2l1, Runx2, and Klf7 were identified (**Figure 4B**). Based on these findings, a transcriptional regulatory network that illustrates the interactions among these transcription factors and their corresponding target genes was constructed, thereby providing insight into the regulatory mechanisms that govern Th2 cell polarization (**Figure 4C**).

### Validation of Gata3 and Pparg as key regulators in Dex-mediated suppression of Th2 responses

**Figure 5:**
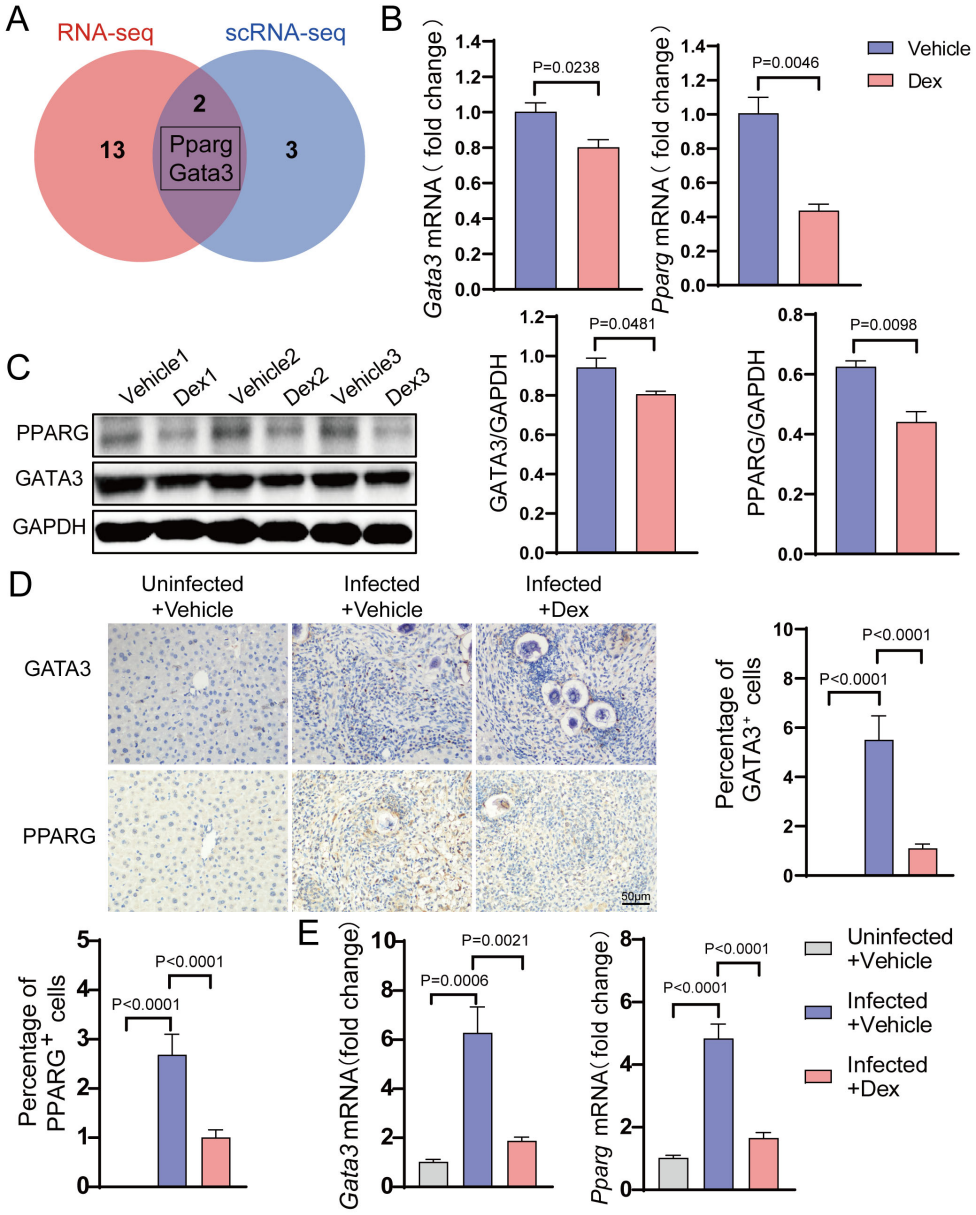
GR activation suppresses key Transcription Factors. **(A)** Venn diagram. **(B)** mRNA expression levels of Gata3 and Pparg in Th2 cells (n=3). **(C)** Protein expression levels of Gata3 and Pparg in Th2 cells (n=3). **(D)** Gata3 and Pparg immunohistochemistry staining (n=6). 400X magnification, scale bar = 50μm. **(E)** mRNA expression levels of Gata3 and Pparg in the liver (n=6). Data are presented as mean ± SD.

To investigate the specific transcription factors that inhibit egg-induced Th2 responses upon GR activation, single-cell RNA-seq data were integrated with transcriptome sequencing. This method identified two important transcription factors, namely Gata3 and Pparg (**Figure 5A**). To validate their roles, their expression was detected in Th2 cells *in vitro*. The results showed that their expression levels were significantly lower in the Dex-treated group compared to the control group (**Figures 5B, C**). Further examination of the liver tissue *in vivo* displayed that, compared with the Uninfected + Vehicle group, the expression levels of Gata3 and Pparg were significantly higher in the Infected + Vehicle group but were markedly lower in the Infected + Dex group (**Figure 5E**). Similarly, compared with the Uninfected + Vehicle group, the number of Gata3 and Pparg-positive cells was significantly higher in the Infected + Vehicle group, while the number of positive cells was significantly lower in the Infected + Dex group (**Figure 5D**). These results conjointly indicate that GR activation significantly inhibits Th2 cell function by down-regulating the expression of Gata3 and Pparg, thereby inhibiting the Th2 responses in schistosomiasis.

## Discussion

Schistosomiasis is a major global health burden, with its pathology primarily attributed to the host’s immune response to schistosome eggs, especially the Th2 response. Herein, GR activation significantly inhibited pathological Th2 responses in schistosomiasis. This inhibition is mediated through the downregulation of key transcription factors Gata3 and Pparg, which play a decisive role in the expression of type 2 cytokines in Th2 cells.

The egg antigen-induced Th2 response is the predominant feature of the late stages of schistosomiasis and is also the chief trigger for the development of egg granulomas and hepatic fibrosis (36, 37). Following the deposition of schistosome eggs in the liver, the egg antigen-induced Th2 response is activated, resulting in the secretion of type 2 effector cytokines such as IL-4, IL-5, and IL-13 (38). These type 2 effector cytokines play a central role in modulating immune responses, promoting schistosome clearance, protecting host tissues, and promoting egg-induced granuloma formation (39). Mice deficient in these cytokines experience accelerated mortality due to their inability to mount an effective type 2 immune response (40, 41). However, a sustained Th2 response can exacerbate disease pathology by stimulating the formation of egg-induced granulomas and hepatic fibrosis in schistosomiasis. Therefore, suppression of Th2 immune responses may represent a promising therapeutic strategy to mitigate the progression of schistosomiasis. Glucocorticoids, as GR agonists, are extensively used for the clinical treatment of inflammatory and autoimmune diseases owing to their powerful anti-inflammatory and immunosuppressive effects. However, their role in modulating egg antigen-induced Th2 immunity in schistosomiasis remains to be elucidated. In the current study, GR activation significantly reduced hepatic granuloma size and fibrosis scores in S.japonicum-infected mice. Furthermore, it down-regulated the expression of type 2 cytokines, including IL-4, IL-5, and IL-13. This may arise from GR activation inhibiting the Th2 response, which can limit the production of these cytokines, thereby decreasing the infiltration level of inflammatory cells and the formation of granulomas.

Th2 cells orchestrate type 2 immune responses by secreting various cytokines, including IL-4, IL-5, and IL-13 (42). Herein, GR activation significantly inhibited the translation and secretion of type 2 cytokines in Th2 cells *in vitro*. Furthermore, the molecular mechanism by which GR activation inhibits Th2 cell function was further investigated through RNA sequencing and bioinformatics analysis. Importantly, the findings revealed that GR activation predominantly affects biological processes related to the regulation of leukocyte differentiation, inflammatory response, cytokine production, and immune system processes. Of note, pathway enrichment analysis indicated significant involvement of the NF-kappa B, MAPK, Rap1, and TNF signaling pathways. In addition, through MCODE analysis, gene modules most significantly influenced by GR activation in Th2 cells were identified, with module 1 genes being particularly related to lymphocyte differentiation. Besides, transcription factor analysis uncovered that 15 key transcription factors could mediate the regulatory effects of GR on genes in module 1. These findings provide novel insights into the molecular mechanisms by which GR modulates Th2 cell function, thereby expanding our understanding of the mechanisms by which GR activation inhibits Th2 responses.

Transcription factors are indispensable molecules that regulate cellular biological functions, gene expression, and cell differentiation processes (43). Additionally, they play a cardinal role in the polarization and function of Th2 cells. In this study, single-cell sequencing data from livers of S.japonicum-infected mice were analyzed, leading to the identification of five key transcription factors that may be critical for regulating Th2 responses in schistosomiasis, namely Gata3, Pparg, Nfe2l1, Runx2, and Klf7. Recent studies have reported that the anti-inflammatory effects of glucocorticoids are primarily mediated by the inhibition of pro-inflammatory transcription factors (44, 45). Moreover, single-cell RNA-seq data were integrated with RNA-sequencing to investigate specific transcription factors that inhibit Th2 cell function upon GR activation (46). Our results revealed two important transcription factors: Gata3 and Pparg. The former is essential for directing the differentiation of naive CD4^+^ T cells towards the Th2 lineage and directly promotes the expression of Th2 cytokines by binding to their promoter regions and facilitating chromatin remodeling (47–50), whilst the latter functions as the primary regulator of adipocyte differentiation and is a significant modulator of lipid metabolism. However, previous studies have documented that Pparg promotes Th2 responses, particularly in conditions such as asthma and obesity (51, 52). It is worthwhile also acknowledging that Pparg influences the expression of Th2-related cytokines and Gata3, thereby enhancing Th2 differentiation (53–55). Our results showed that the expression levels of both Gata3 and Pparg were significantly elevated in the liver tissues of *S.japonicum*-infected mice but were markedly reduced following Dex treatment. Likewise, *in vitro* studies demonstrated a decrease in the expression levels of Gata3 and Pparg in Th2 cells treated with dexamethasone. Overall, these findings suggest that GR activation suppresses egg antigen-induced Th2 responses by downregulating Gata3 and Pparg, which are critical regulators of Th2 cell function.

Despite providing novel insights into the mechanism by which GR activation inhibits Th2 immune responses in schistosomiasis via the downregulation of Gata3 and Pparg, this study has several limitations. First, our findings are primarily based on animal models and *in vitro* experiments. While these models provide robust evidence for the suppressive effect of GR activation on Th2 immunity, their direct relevance to human schistosomiasis requires further validation. Second, although RNA sequencing and single-cell RNA sequencing were employed to analyze GR-mediated transcriptional changes in Th2 cells, these analyses were conducted at specific time points, which may not fully capture the dynamic regulatory landscape during disease progression. Moreover, while Gata3 and Pparg were identified as key transcriptional targets of GR activation, other transcription factors or signaling pathways may also contribute to Th2 modulation, necessitating further investigation. Lastly, although this study focused on the inhibitory effects of GR activation on Th2 immune responses, its potential off-target effects on other immune cell populations and broader immunological consequences remain to be elucidated. Future research should explore strategies to mitigate potential adverse effects while maximizing the therapeutic benefits of GR activation in schistosomiasis.

In summary, this study demonstrates that GR activation suppresses the expression of type 2 cytokines by downregulating the transcription factors Gata3 and Pparg, thereby inhibiting Th2 responses in schistosomiasis. These findings highlight the potential of targeting GR to alleviate Th2-related pathology in schistosomiasis and suggest a possible complementary therapeutic strategy for disease management.

## Data Availability

The datasets presented in this study can be found in online repositories. The names of the repository/repositories and accession number(s) can be found below: CRA020304 (GSA; https://ngdc.cncb.ac.cn/gsa/s/b9568Bp1.
