# Correction: Glucocorticoid receptor inhibits Th2 immune responses by down-regulating Pparg and Gata3 in schistosomiasis

**DOI:** 10.3389/fimmu.2026.1875583

**Published:** 2026-05-15

**Authors:** Tao Sun, Xiaojuan Bi, Ning Yang, Xue Zhang, Jin Chu, Liang Li, Hui Liu, Rui Tang, Renyong Lin

**Affiliations:** 1 State Key Laboratory of Pathogenesis, Prevention and Treatment of High Incidence Diseases in Central Asia, Clinical Medical Research Institute, The First Affiliated Hospital of Xinjiang Medical University, Urumqi, Xinjiang, China; 2 Department of Tropical Infectious Diseases, Naval Medical University, Shanghai, China

**Keywords:** glucocorticoid receptor, schistosomiasis, Pparg, Gata3, Th2 immune responses

The **Author contributions** statement was erroneously given as “TS: Investigation, Project administration, Writing - original draft, Conceptualization. XB: Writing - review & editing, Data curation, Formal analysis. NY: Writing - review & editing, Project administration, Supervision, Visualization. RL: Funding acquisition, Supervision, Writing - review & editing. RT: Project administration, Writing - review & editing, Formal analysis, Funding acquisition, Supervision. XZ: Project administration, Writing - review & editing. JC: Project administration, Writing -review & editing, Formal analysis, Software. LL: Writing - review &editing, Investigation, Project administration, Validation. HL: Writing - review & editing.” The correct author contributions statement is “TS: Investigation, Project administration, Writing - original draft, Conceptualization. XB: Writing - review & editing, Data curation, Formal analysis. NY: Writing - review & editing, Project administration, Supervision, Visualization. RL: Supervision, Writing - review & editing. RT: Project administration, Writing - review & editing, Formal analysis, Funding acquisition, Supervision. XZ: Project administration, Writing - review & editing. JC: Project administration, Writing - review & editing, Formal analysis, Software. LL: Writing - review &editing, Investigation, Project administration, Validation. HL: Writing - review & editing.”

Futhermore an incorrect **Funding** statement was provided. The correct **Funding** statement reads:

“The author(s) declare that financial support was received for the research and/or publication of this article. This work was supported by the Program for Clinical Research in the Health Industry of Shanghai Municipal Health Commission (20224Y0103).”

And finally we need to add a new **Acknowledgments** section with the following content: “The authors acknowledge the National Natural Science Foundation of China (82060371) for supporting the establishment of experimental platforms and providing the scientific hypotheses that inspired this work. The authors acknowledge the Xinjiang Tianshan Project (2022TSYCLJ0032), Key Program of Xinjiang Natural Science Foundation (2022D01D59), National Natural Science Foundation of China (32060223), Xinjiang Science Foundation for Distinguished Young Scholars (2022D01E67), Plan for Supporting Xinjiang through Science and Technology in Xinjiang Uygur Autonomous Region (2024E02040) and Open Topics of State Key Laboratory of Pathogenesis, Prevention, and Treatment of Central Asian High Incidence Diseases(SKL-HIDCA-2024-19) for their support in the development of experimental techniques for this study. The research reported here was conducted independently and supported by other funding sources.”

The original version of this article has been updated.

