# Supplementary figures and images for: Glucocorticoid receptor inhibits Th2 immune responses by down-regulating Pparg and Gata3 in schistosomiasis
Source: Front Immunol. 2025 Mar 24;16:1518586. doi: 10.3389/fimmu.2025.1518586 (PMC11973390; doi:10.3389/fimmu.2025.1518586)

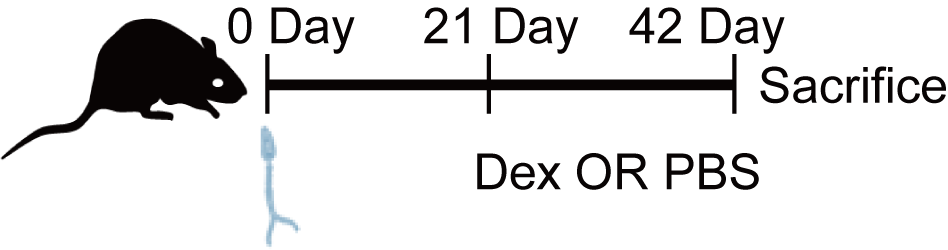

Supplement: Supplementary file 1 [file Image1.tif]

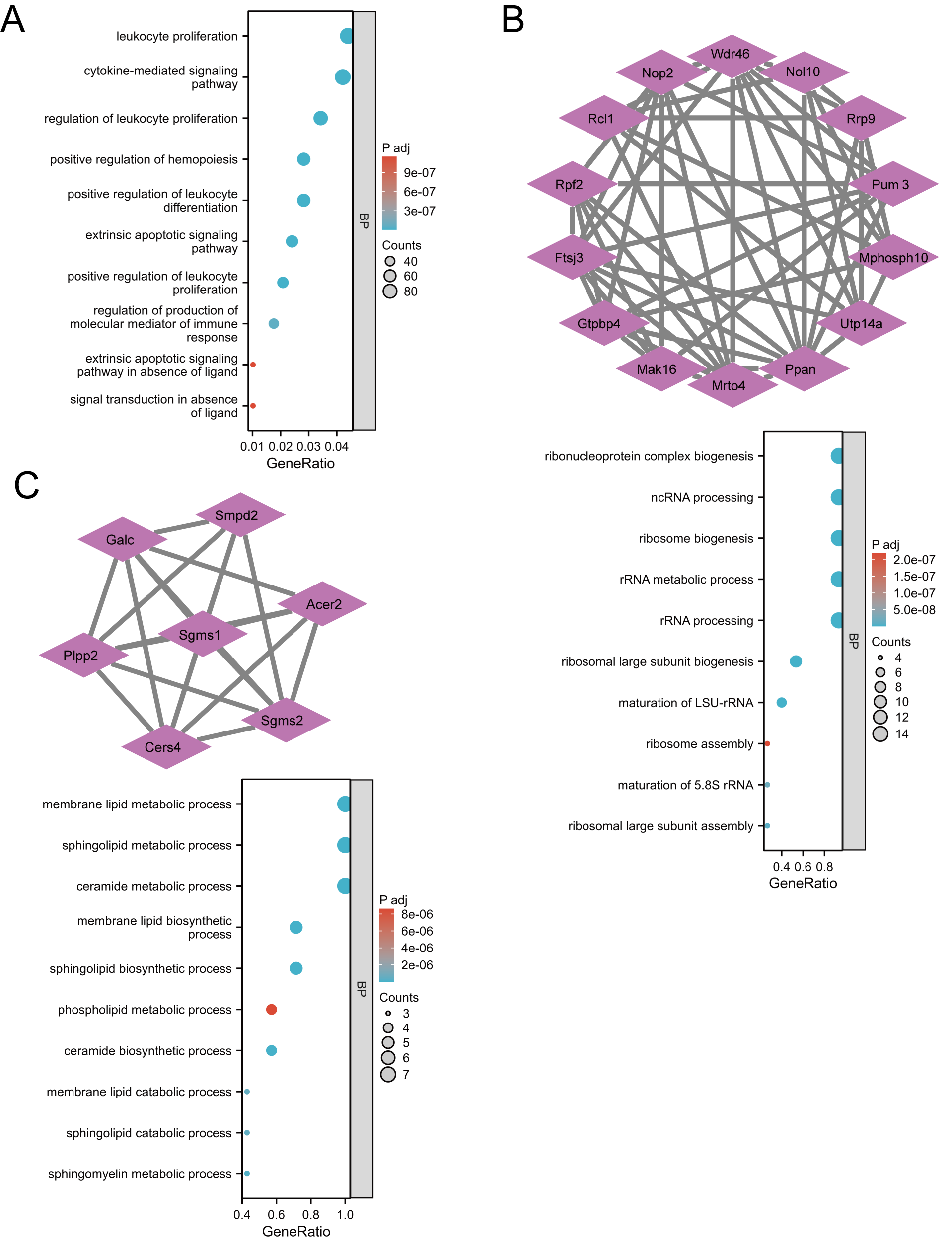

Supplement: Supplementary file 2 [file Image2.tif]

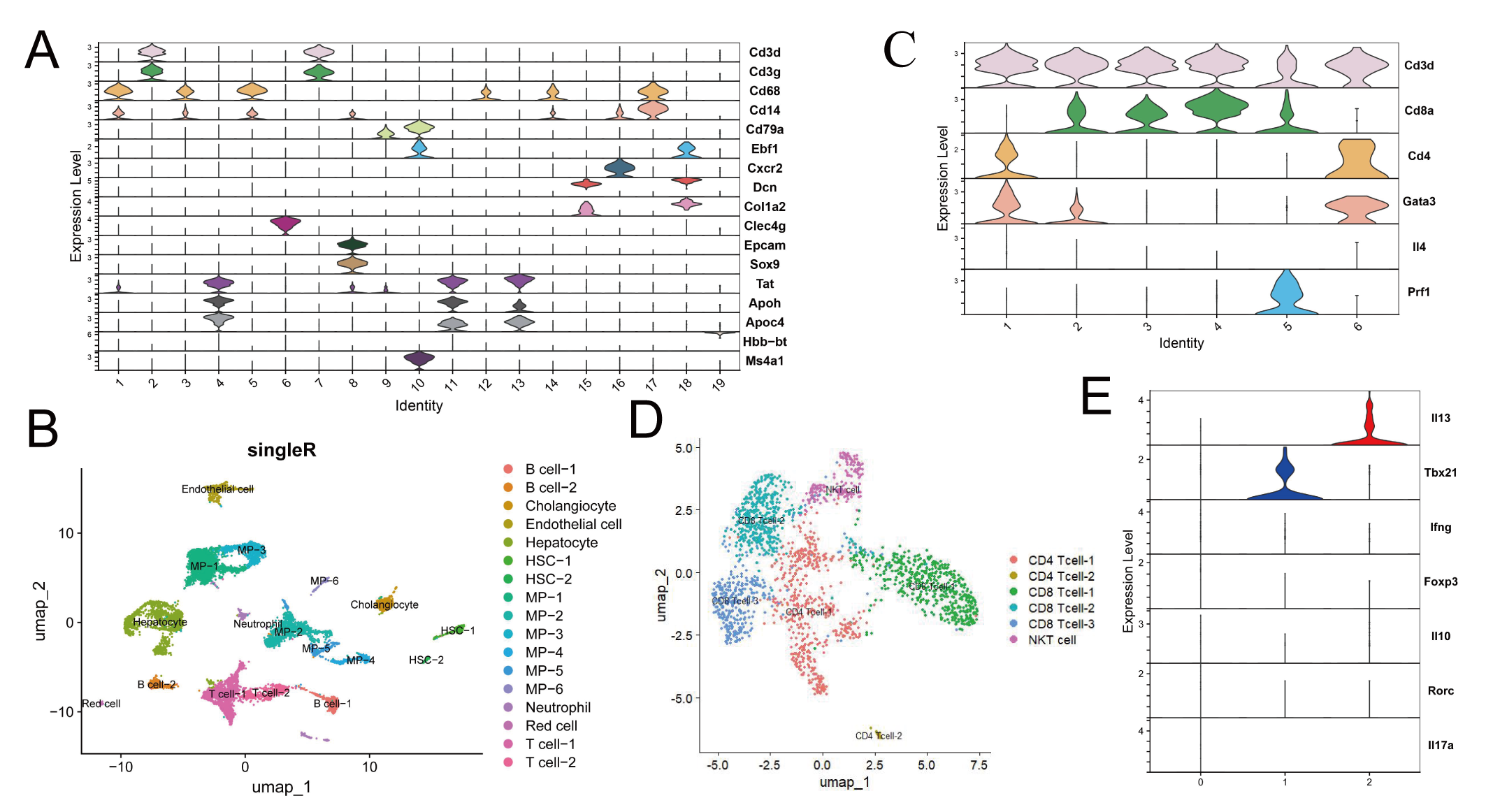

Supplement: Supplementary file 3 [file Image3.tif]

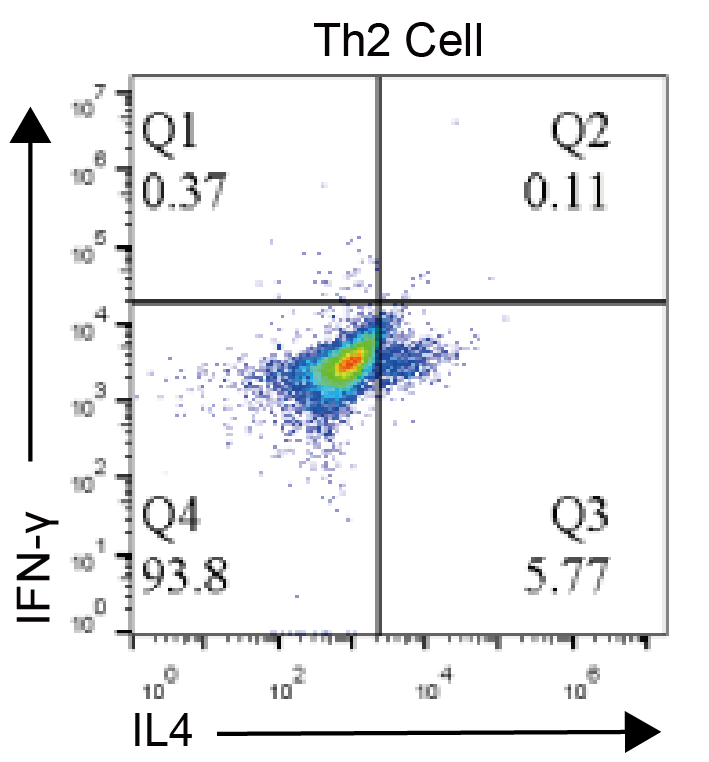

Supplement: Supplementary file 4 [file Image4.tif]
