# Supplementary material for: Glucocorticoid receptor inhibits Th2 immune responses by down-regulating Pparg and Gata3 in schistosomiasis
Source: Front Immunol. 2025 Mar 24;16:1518586. doi: 10.3389/fimmu.2025.1518586 (PMC11973390; doi:10.3389/fimmu.2025.1518586)
Supplement: Supplementary file 6 [file Table2.docx]

Supplementary Material

**Table S1 Primers used in this study**

| Gene | Forward primer | Reverse primer |
| --- | --- | --- |
| Il13 | CCTGGCTCTTGCTTGCCTT | GGTCTTGTGTGATGTTGCTCA |
| Il4 | GGTCTCAACCCCCAGCTAGT | GCCGATGATCTCTCTCAAGTGAT |
| Il5 | CTCTGTTGACAAGCAATGAGACG | TCTTCAGTATGTCTAGCCCCTG |
| Gata3 | CTCGGCCATTCGTACATGGAA | GGATACCTCTGCACCGTAGC |
| Pparg | TCGCTGATGCACTGCCTATG | GAGAGGTCCACAGAGCTGATT |
| Gapdh | TGACCTCAACTACATGGTCTACA | CTTCCCATTCTCGGCCTTG |

Figure S1 Time course of the experimental groups.

Figure S2 Significant gene modules of DEGs. A GO enrichment analysis of genes in module 1. B-C PPI network of genes and GO enrichment analysis of genes in the clustering modules 2 and 3.

Figure S3 Identification of cell types from scRNA-seq data. A Expression levels of marker genes in different clusters of the liver. B UMAP plot of annotation clusters of the liver. C Expression levels of marker genes in different subclusters of T cells in the liver. D UMAP plot of annotation clusters of T cells. E Expression levels of marker genes in different subclusters of CD4 T cells.

Figure S4 Dot plot showing the gating strategy based on IFN-γ and IL-4 in induced Th2 cells (n=3).
